# Dietary Inflammatory Potential and Cardiometabolic Risk Factors in School‐Aged Adolescents

**DOI:** 10.1111/ijpo.70133

**Published:** 2026-07-02

**Authors:** Luana de Oliveira Leite, Mônica Leila Portela de Santana, Nadjane Ferreira Damascena, Jacqueline Costa Dias Pitangueira, Priscila Ribas de Farias Costa

**Affiliations:** ^1^ School of Nutrition Federal University of Bahia (UFBA) Salvador Bahia Brazil; ^2^ Health Sciences Center Federal University of Recôncavo da Bahia (UFRB) Santo Antônio de Jesus Bahia Brazil

**Keywords:** adolescents, blood pressure, dietary inflammatory index, obesity

## Abstract

**Background:**

The Dietary Inflammatory Index (DII) and its version adapted for children (C‐DII) have emerged as promising tools to estimate the inflammatory potential of the diet and to enhance the understanding of the relationship between subclinical chronic inflammation and cardiometabolic risk during adolescence.

**Objective:**

To investigate the associations of DII and C‐DII with cardiometabolic risk indicators in adolescent students.

**Methods:**

This cross‐sectional study was conducted with 552 adolescents aged 10–17 years (66% girls; mean age: 13.6 ± 1.7 years). DII scores ranged from −1.34 to 5.04 (median: 2.62) and C‐DII scores ranged from −1.99 to 4.39 (median: 1.96) and were categorised as anti‐inflammatory diets (below the median) and pro‐inflammatory diets (above the median). Outcomes included body mass index (BMI), waist circumference (WC), waist‐to‐height ratio (WHtR) and blood pressure. Multilevel linear regression was applied.

**Results:**

Adolescents with a pro‐inflammatory diet (above the C‐DII median) had a higher mean BMI (*β* = 0.97 kg/m^2^; 95% CI: 0.02; 1.92). Among girls, higher mean BMI values were observed for both DII (*β* = 1.29 kg/m^2^) and C‐DII (*β* = 1.38 kg/m^2^). Among those > 14 years, pro‐inflammatory diets were associated with higher BMI (*β* = 2.16 kg/m^2^), WC (*β* = 3.73 cm) and WHtR (*β* = 0.03) for both indices. No associations were observed with blood pressure.

**Conclusion:**

Pro‐inflammatory diets were positively associated with higher adiposity, especially among girls and older adolescents. Early dietary interventions may help reduce lifetime cardiometabolic risk.

## 1 Introduction

The increasing prevalence of obesity and other cardiometabolic risk factors, such as dyslipidemia, insulin resistance and hypertension, among adolescents has become a growing public health concern, particularly in low‐ and middle‐income countries such as Brazil [1, 2]. These risk factors are frequently associated with a state of low‐grade chronic inflammation, characterised by persistently elevated concentrations of pro‐inflammatory cytokines, including interleukin‐6 (IL‐6), tumour necrosis factor‐alpha (TNF‐α) and C‐reactive protein (CRP) [3, 4].

Evidence indicates that diet plays a central role in modulating subclinical chronic inflammation through the intake of nutrients and bioactive compounds with pro‐ or anti‐inflammatory properties [5, 6, 7]. Dietary patterns characterised by high energy density, low fibre content and elevated consumption of simple sugars, saturated fats and sodium promote inflammatory responses, contributing to adipose tissue accumulation and metabolic dysfunction, including alterations in lipid and glucose metabolism and increased blood pressure [8, 9, 10]. This scenario is particularly concerning among adolescents, who are often exposed to unhealthy dietary patterns marked by high consumption of soft drinks, fast food, processed meats and ultra‐processed foods, along with low intake of fruits, vegetables, whole grains and minimally processed foods [9, 11, 12, 13].

To address the inflammatory potential of diet, the Dietary Inflammatory Index (DII) was developed as a tool to quantify the pro‐ or anti‐inflammatory properties of an individual's diet, based on the intake of 45 dietary parameters and their effects on six inflammatory biomarkers [14]. The DII has been widely applied in adult populations and has shown associations with multiple health outcomes, including obesity, insulin resistance, dyslipidemia, hypertension and cardiovascular risk [15, 16, 17, 18].

The DII has also been validated for use in paediatric populations [19] and subsequently applied in various adolescent groups [20, 21, 22, 23], including Brazilian adolescents [24]. However, given that the index was originally developed using adult dietary data, an adapted version for children and adolescents was needed, excluding items less commonly consumed by this age group, such as black and green tea, turmeric, garlic, ginger and specific flavonoids [25]. Accordingly, in 2018, Khan et al. proposed the Children's Dietary Inflammatory Index (C‐DII), which includes only 25 of the original 45 dietary parameters. This adaptation aims to better capture the dietary reality of paediatric populations [25].

Nevertheless, the C‐DII still presents important limitations. The database used for its construction remains adult‐based, potentially compromising its validity in children. Moreover, there is a scarcity of studies directly validating the C‐DII across different age groups, pubertal stages and cultural contexts [26, 27]. These limitations highlight the need to comparatively assess the performance of the DII and C‐DII in studies involving children and adolescents to determine which tool is most suitable for detecting associations between pro‐inflammatory dietary patterns and cardiometabolic risk factors in this population, especially in middle‐income countries, where dietary patterns and socioeconomic conditions may amplify vulnerability. To the best of our knowledge, this is the first Brazilian study to apply the C‐DII in adolescents, providing a pioneering assessment of its utility in this population context.

Adolescence is a critical developmental period, characterised by substantial physiological, behavioural and nutritional changes that may have long‐term effects into adulthood [28]. Early identification of environmental factors, such as diet, that negatively influence cardiometabolic health is therefore essential for planning effective preventive interventions. Accordingly, the objective of this study was to evaluate the associations between the DII and C‐DII and cardiometabolic risk indicators, including body adiposity and blood pressure, in a sample of school‐aged adolescents from a major Brazilian city.

## 2 Methods

### 2.1 Study Design and Population

This is a cross‐sectional study conducted with 552 adolescents aged 10 to 17 years, enrolled in grades 6 through 9 in public state schools in Salvador, Bahia, one of the largest capitals in northeastern Brazil. The study used baseline data from a larger research project entitled ‘Influence of Overweight and School Environment on Cardiovascular Risk Factors in Adolescents: A Prospective Approach’, which is part of the multi‐country study ‘School Policies and Cardiovascular Risk: A Multi‐Country Study’.

Participants were recruited from six schools under the Regional Education Coordinations (CREs) of the municipality of Salvador, Bahia. All students from these schools were invited to participate and received an invitation letter addressed to their parents or legal guardians. School selection was conducted by convenience, based on similarities with institutions included in the multi‐country study and the interest of school administrators in participating in the project. The final sample comprised two military schools and four state schools located in different neighbourhoods of Salvador, Brazil.

Adolescents aged 10 to 19 years who were enrolled in the selected schools and had parental and/or guardian consent were eligible for inclusion. Participants using medications known to affect cardiometabolic markers (e.g., anticonvulsants, thiazide diuretics, corticosteroids, among others) or with a medical diagnosis of diabetes mellitus, chronic kidney disease, liver disease, or hypothyroidism, conditions that influence plasma concentrations of these markers, were excluded. Additional exclusion criteria included pregnancy, lactation and physical disabilities that prevented accurate anthropometric assessment.

Given that the original project did not account for the parameters addressed in this study, post hoc sample power was calculated. Assuming a significance level (*α*) of 0.05 and an estimated effect size (Cohen's *f*) of 0.15, statistical power was determined to be 91% for the variables obesity, abdominal obesity and blood pressure, indicating adequate power to detect effects of moderate magnitude [29, 30].

This study was approved by the Research Ethics Committee of the School of Nutrition, Federal University of Bahia (approval number 1 139 343, July 6, 2015; CAAE: 42053014.0.0000.5023). Written informed consent was obtained from parents and/or legal guardians and written assent was provided by all participating adolescents.

### 2.2 Data Collection

Socioeconomic, demographic, lifestyle, clinical, anthropometric and dietary intake data were collected by a trained research team. All baseline measurements were obtained between 2016 and 2018.

### 2.3 Socioeconomic, Demographic and Lifestyle Information

These data were self‐reported by the adolescents and recorded using a structured questionnaire, except for socioeconomic characteristics, which were provided by parents and/or legal guardians. Demographic variables included sex, skin colour (race) and age. Skin colour or race was categorised according to the Brazilian Institute of Geography and Statistics (IBGE, 2022), using the classifications employed to characterise the Brazilian population: White, Black, Brown (Pardo), Yellow (Asian descent) and Indigenous. These categories were based on self‐identification [31].

Socioeconomic status was assessed using the Critério de Classificação Econômica Brasil (Brazilian Economic Classification Criterion—CCEB), which estimates the purchasing power of individuals and households in urban settings. This criterion does not aim to define ‘social classes’, but rather to group individuals into market‐based economic strata [32]. The total score ranges from 0 to 100 points and is divided into six categories: A (45–100 points), B1 (38–44 points), B2 (29–37 points), C1 (23–28 points), C2 (17–22 points) and D/E (0–16 points). For the present study, individuals were grouped into three socioeconomic categories: A–B (high), C (intermediate) and D–E (low).

Lifestyle variables included physical activity, alcohol consumption and smoking. Physical activity level was assessed using the Physical Activity Questionnaire for Adolescents (PAQ‐A), developed by Kowalski et al. [33] and previously validated in Brazil by Matsudo et al. [34]. The PAQ‐A consists of eight items scored on a scale from 1 (low activity) to 5 (high activity), used to calculate the adolescent's overall physical activity score. The questionnaire evaluates regular physical activity during leisure time, at school and in recreational settings over the past 7 days. Based on the total score, adolescents were classified as sedentary, low active, moderately active, active, or very active [33].

### 2.4 Anthropometric Measurements

Anthropometric assessment (weight, height and waist circumference [WC]) was conducted by a trained team following standardised techniques described in the literature [35]. Weight was measured using a portable digital scale (Filizola, capacity 150 kg, precision 100 g). Height was measured with a Leicester Height Measure stadiometer, with readings recorded to the nearest millimetre, according to recommended procedures. WC was measured using a non‐elastic fibreglass tape with centimetre markings [35].

All measurements were taken in duplicate and the mean of the two readings was used as the final value and recorded in the corresponding questionnaire. If the discrepancy between measurements exceeded the recommended precision (0.5 cm for WC and 0.1 cm for height), a third measurement was performed and the mean of the two closest readings was used [35].

Body mass index (BMI) was calculated as weight (kg) divided by height squared (m^2^) [BMI = W (kg)/H^2^ (m)], using the World Health Organization growth charts as reference [36] for individuals aged 5–19 years, which account for sex and age. Given the lack of consensus regarding WC cut‐off points for children and adolescents, the 90th percentile of the study sample was adopted to classify abdominal fat excess, as proposed by Freedman et al. [37]. The waist‐to‐height ratio (WHtR) was calculated as WC (cm) divided by height (cm), with values ≥ 0.50 used to define abdominal obesity [38].

### 2.5 Blood Pressure

Blood pressure (BP) was measured using a digital sphygmomanometer (Omron HEM 705 CP), suitable for the participants' age group and arm circumference. The measurement procedure followed the recommendations of the VII Brazilian Guidelines on Hypertension. Prior to BP assessment, participants were screened to ensure compliance with pre‐measurement recommendations, including no physical activity within 60 min, bladder emptying and no intake of coffee or stimulant foods within 30 min before the exam. Measurements were taken in triplicate, with a 3‐min interval between readings and the mean of the three measurements was used as the final value and recorded in the respective questionnaire [39].

### 2.6 Dietary Assessment

Adolescents' dietary intake was assessed. Information was provided directly by participants, with the support of trained interviewers to enhance data reliability. A photographic album depicting various food and beverage portion sizes was used to assist participants in estimating their consumed portions [40]. Two non‐consecutive 24‐h dietary recalls were applied, with a minimum interval of 7 days and a maximum of 45 days, intentionally covering one typical day and one atypical day. Habitual intake was estimated using the Multiple Source Method (MSM), a recommended approach to adjust for within‐person variability in population‐based dietary studies [41].

Portion sizes reported in the 24‐h recalls were standardised using food composition tables and household measurement manuals [42, 43, 44], including recommendations by Fisberg and Villar [45]. All common units for each food item were converted into grams, milligrams, litres, or millilitres to assess daily energy (kcal) and nutrient intake. Energy and nutrient contents were determined using the Nutri Virtual Program, developed by the Department of Nutrition at the University of São Paulo [46]. Culinary preparations not present in reference tables or the software database were adapted from online recipes and subsequently incorporated into the standardisation list.

Participants with extreme energy intake values (< 800 or > 7000 kcal/day for boys and < 600 or > 5000 kcal/day for girls) were excluded, as recommended by Willett [47]. These cut‐offs are widely used in epidemiological studies, including in the Brazilian context, to ensure the biological plausibility of dietary intake data.

### 2.7 Dietary Inflammation Indices

Using dietary data derived from the two 24‐h recalls, the methodology proposed by Shivappa et al. [14] and Khan et al. [25] was applied to calculate the Dietary Inflammatory Index (DII) and the Children's Dietary Inflammatory Index (C‐DII), respectively, for each adolescent.

Briefly, the DII calculation was based on individual dietary intake data and linked to a global database providing robust estimates of the mean and standard deviation for each dietary parameter. A *Z*‐score was computed to indicate an individual's exposure relative to the global mean by subtracting the global mean and dividing by the standard deviation for each parameter [14].

To minimise right‐skewness, the *Z*‐score was converted to a percentile score. For a symmetric distribution centred on 0 and bounded between −1 and +1, each centred percentile score was calculated by doubling the percentile and subtracting 1. This centred percentile score was then multiplied by the respective parameter‐specific inflammatory effect score to obtain the DII score for each dietary parameter. All parameter‐specific DII scores were summed to generate the total DII score for each participant [14]. Similarly, the C‐DII was calculated using dietary parameters relevant to the paediatric population [25].

In this study, 28 of the 45 original DII parameters were used (vitamin B12, carbohydrates, cholesterol, calories, total fat, iron, protein, saturated fat, trans fat, vitamin B6, fibre, folate, garlic, magnesium, monounsaturated fat, vitamin B3, onion, polyunsaturated fat, vitamin B2, selenium, vitamin B1, vitamin A, vitamin C, vitamin E, vitamin D, zinc, pepper, oregano) and 23 of the 25 parameters used by Khan et al. were included (vitamin A, thiamine, riboflavin, niacin, vitamin B6, folate, vitamin B12, vitamin D, vitamin C, vitamin E, energy, carbohydrates, fibre, total fat, saturated fat, monounsaturated fatty acids [MUFA], polyunsaturated fatty acids [PUFA], cholesterol, protein, iron [Fe], magnesium [Mg], selenium [Se], zinc [Zn]). The remaining parameters from the original DII and C‐DII were excluded due to either non‐consumption by participants or lack of information in the Nutri Virtual Program [20, 21, 22, 23, 48].

To control for energy intake, DII was calculated per 1000 kcal of consumed food [25]. Higher DII scores indicate a greater pro‐inflammatory potential of the diet, while lower scores indicate a greater anti‐inflammatory potential [14].

### 2.8 Variable Identification

#### 2.8.1 Primary Exposure Variables

The primary exposure variable was the dietary inflammatory potential, incorporated into the models as a categorical variable. The median of the study sample was used as the cut‐off to dichotomise DII and C‐DII scores, with values below the median serving as the reference category (0; anti‐inflammatory diet) and values above the median representing the risk category (1; pro‐inflammatory diet).

#### 2.8.2 Outcome Variables

The outcome variables were BMI, WC, waist‐to‐height ratio (WHtR), systolic blood pressure (SBP) and diastolic blood pressure (DBP). These variables were included in the models as continuous variables. For descriptive purposes, they were categorised as follows: BMI—no excess weight (underweight and normal weight = 0) and excess weight (overweight and obesity = 1); WC—below the 90th percentile (no abdominal obesity = 0) and ≥ 90th percentile (abdominal obesity = 1); WHtR—≤ 0.50 (no abdominal obesity = 0) and > 0.50 (abdominal obesity = 1).

#### 2.8.3 Covariates

The models were adjusted for the following covariates: age (continuous), sex (0 = male; 1 = female), skin colour/race (0 = white; 1 = mixed race [parda]; 2 = black; 3 = other), socioeconomic status (0 = high, 1 = intermediate, 2 = low), physical activity (0 = sedentary or lightly active, 1 = moderately active, 2 = active or highly active), smoking status (0 = no; 1 = yes), alcohol consumption (0 = no; 1 = yes) and total energy intake (continuous).

### 2.9 Data Analysis

Data were entered into EPI‐INFO. Descriptive analyses were conducted by presenting continuous variables as means and standard deviations and categorical variables as absolute and relative frequencies. Sample normality was assessed using the Kolmogorov–Smirnov test. Socioeconomic, demographic, lifestyle, anthropometric and clinical variables were compared between categories below and above the median of the respective indices using Pearson's chi‐square test for categorical variables and Student's *t*‐test for continuous variables. Dietary intake characteristics were also compared across index categories using Student's *t*‐test.

To investigate the relationship between dietary inflammatory potential (assessed by DII and C‐DII) and the outcomes of interest (BMI, WC, WHtR, systolic BP and diastolic BP), multilevel linear regression models were calculated in both crude and adjusted forms. The multilevel approach was justified by the hierarchical structure of the data, as participants were nested within different schools. Such clustering can generate variability in outcomes both within and between schools, making multilevel modelling necessary to properly account for this dependence structure and avoid biased estimates of dietary effects [48].

Adjusted models included covariates identified in previous studies as potential confounders [19, 20, 21, 22, 23, 49], namely: age, sex, skin colour, socioeconomic status, smoking and alcohol history and physical activity. Interaction terms between independent variables and outcomes were tested, revealing significant interactions for sex (*p* for interaction = 0.01) and age (≤ 14 years vs. > 14 years; *p* for interaction = 0.02); *p* for interaction < 0.05 indicates a statistically significant interaction.

Age was stratified into ≤ 14 years and > 14 years, considering that this cutoff point roughly represents the transition between early adolescence and middle/late adolescence, a period marked by important changes related to pubertal maturation, body composition and cardiometabolic factors [50, 51]. Potential effect modification by age was initially assessed by including interaction terms in the multilevel regression models, with age treated as a continuous variable. Interaction significance was evaluated using likelihood ratio tests comparing models with and without the interaction term. When a statistically significant interaction was identified, age was categorised into ≤ 14 years and > 14 years and stratified analyses were subsequently performed to facilitate interpretation of the findings. Therefore, analyses were stratified by these variables to assess the magnitude and direction of associations within each subgroup. Results of the multilevel linear regression analyses were reported as *β* coefficients with 95% confidence intervals.

Analyses were conducted using R software with the lme4 package for multilevel model fitting. Models were estimated using the lmer() function and a significance level of 5% was adopted for all tests.

## 3 Results

### 3.1 Population Characteristics and Nutrient Intake by DII and C‐DII Categories

The study sample comprised 552 adolescents from Salvador (BA), Brazil, after excluding six participants with extreme energy intake values. The mean age was 13.6 years (SD = 1.69), with the majority aged ≤ 14 years (60%) and a predominance of females (66%). DII scores ranged from −1.34 to 5.04, with a mean of 2.44 and a median of 2.62, whereas C‐DII scores ranged from −1.99 to 4.39, with a mean of 1.78 and a median of 1.96.

Significant associations were observed between DII categories and sex, age and skin colour, with higher frequencies of females (71.7%), participants aged ≤ 14 years (67%) and mixed‐race (parda) adolescents (44%) in the above‐median DII category. Similar results were found for the C‐DII, with significant associations for sex (*p* = 0.001), age (*p* < 0.001) and skin colour (*p* = 0.042) between the above‐median (more pro‐inflammatory diet) and below‐median (more anti‐inflammatory diet) categories. No significant differences were observed between DII or C‐DII categories and socioeconomic status, physical activity, smoking, or alcohol consumption (Table 1).

**TABLE 1:** Sociodemographic characteristics and cardiometabolic risk indicators according to DII and C‐DII categories (*n* = 552) among school‐aged adolescents in Salvador, Bahia, Brazil, 2016–2018.

Characteristics	Total *N* (%)	DII			C‐DII		
		Below the median (≤ 2.62) (*n* = 276)	Above the median (> 2.62) (*n* = 276)	*p* *	Below the median (≤ 1.96) (*n* = 276)	Above the median (> 1.96) (*n* = 276)	*p* *
Sex (%)							
Female	362 (66.0)	164 (59.4)	198 (71.7)	0.002	163 (59.0)	199 (72.1)	0.001*
Male	190 (34.0)	112 (41.5)	78 (28.3)		113 (41.0)	77 (27.9)	
Age (years)	13.62 (1.69)	13.83 (1.79)	13.41 (1.56)	0.003	13.84 (1.78)	13.39 (1.56)	0.002*
Age range (%)							
≤ 14 years	333 (60.0)	147 (53.3)	186 (67)	0.001	146 (52.3)	187 (67.8)	0.000*
> 14 years	219 (40.0)	129 (46.7)	90 (33)		130 (47.1)	89 (32.2)	
Skin colour (%)							
White	45 (08.0)	31 (11.2)	14 (05.1)	0.048	31 (11.2)	14 (05.1)	0.042
Black	146 (26.0)	70 (25.4)	76 (27.5)		70 (25.4)	76 (27.5)	
Brown	228 (41.0)	107 (38.8)	121 (43.8)		106 (38.4)	122 (44.2)	
Others (Yellow/Indigenous)	63 (12.0)	34 (12.3)	29 (10.5)		34 (12.3)	29 (10.5)	
Missing	70 (13.0)	34 (12.3)	36 (13.1)		35 (12.7)	35 (12.7)	
Socioeconomic status (ABEP) (%)							
High (A + B1 + B2)	173 (31)	89 (32.2)	84 (30.5)	0.282	89 (32.2)	84 (30.4)	0.203
Intermediate (C1 + C2)	219 (40.0)	99 (35.9)	120 (43.5)		98 (35.5)	121 (43.8)	
Low (D + E)	46 (08.0)	26 (09.4)	20 (07.2)		26 (09.4)	20 (07.2)	
Missing	114 (21.0)	62 (22.5)	52 (18.8)		63 (22.8)	51 (18.5)	
Physical activity (%)							
Sedentary	76 (14.0)	30 (11.0)	46 (16.7)	0.978	30 (11.0)	46 (16.7)	0.993
Low active	258 (47.0)	111 (40.2)	147 (53.3)		111 (40.2)	147 (53.3)	
Moderately active	112 (20.0)	47 (17.0)	65 (23.5)		47 (17.0)	65 (23.5)	
Active	21 (03.5)	13 (04.7)	8 (02.90)		13 (04.7)	8 (02.9)	
Highly active	3 (00.5)	2 (00.7)	1 (00.4)		2 (00.7)	1 (00.4)	
Missing	82 (15.0)	73 (26.4)	9 (03.2)		73 (26.4)	9 (03.2)	
Smoking (%)							
Yes	5 (01.0)	3 (01.1)	2 (00.7)	0.660	3 (1.1)	2 (00.7)	0.653
No	477 (86.0)	239 (86.6)	238 (86.2)		238 (86.2)	239 (86.6)	
Missing	70 (13.0)	34 (12.3)	36 (13.1)		35 (12.7)	35 (12.7)	
Alcohol consumption (%)							
Yes	52 (09.5)	32 (11.6)	20 (07.2)	0.081	32 (11.6)	20 (07.3)	0.075
No	431 (78.0)	210 (76.1)	221 (80.1)		209 (75.7)	222 (80.4)	
Missing	69 (12.5)	34 (12.3)	35 (12.7)		35 (12.7)	34 (12.3)	
BMI (%)							
Without excess weight	373 (68.0)	198 (71.7)	175 (63.4)	0.048	198 (71.7)	175 (63.4)	0.035
With excess weight	163 (29.0)	71 (25.7)	92 (33.3)		70 (25.4)	93 (33.7)	
Missing	16 (03.0)	7 (02.6)	9 (03.3)		8 (02.9)	8 (02.9)	
WC (%)							
< P90	496 (90.0)	249 (90.2)	247 (89.5)	0.778	248 (89.9)	248 (89.9)	1.000
≥ P90	56 (10.0)	27 (09.8)	29 (10.5)		28 (10.1)	28 (10.1)	
WHtR (%)							
< 0.5	451 (82.0)	229 (83.0)	222 (80.4)	0.741	229 (83.0)	222 (80.4)	0.741
≥ 0.5	90 (16.0)	42 (15.2)	48 (17.4)		42 (15.2)	48 (17.4)	
Missing	11 (02.0)	5 (01.8)	6 (02.2)		5 (01.8)	6 (02.2)	
SBP (mm/Hg)	106 (100.114)	107.10 (12.74)	104.73 (17.43)	0.069	107.06 (12.72)	104.76 (17.45)	0.077
DBP (mm/Hg)	62 (058.67)	62.59 (07.82)	61.83 (10.76)	0.345	62.57 (07.81)	61.84 (10.76)	0.360

*Note:* Data are presented as mean (SD) for continuous variables or *N* (%) for categorical variables.

Abbreviations: BMI, body mass index; C‐DII, children's dietary inflammatory index; DBP, diastolic blood pressure; DII, dietary inflammatory index; *N*, number; SBP, systolic blood pressure; SD, standard deviation; WC, waist circumference; WHtR, waist‐to‐height ratio.

* *p*‐values were calculated for differences between below‐median and above‐median categories of DII and C‐DII using Student's *t*‐test (continuous variables) and Pearson's chi‐square test (categorical variables).

Regarding cardiometabolic outcomes, the prevalence of excess weight (overweight + obesity) was 29%. Abdominal obesity, assessed by waist circumference (WC ≥ 90th percentile), was present in 10% of the sample, while a waist‐to‐height ratio (WHtR ≥ 0.5) indicated central adiposity in 16% of adolescents. Mean systolic blood pressure (SBP) was 106 mmHg (SD = 13.4) and mean diastolic blood pressure (DBP) was 62 mmHg (SD = 8.6). Statistically significant differences were observed only for BMI across DII and C‐DII categories (*p* = 0.048 and *p* = 0.035, respectively), whereas no significant differences were found for the other cardiometabolic indicators (Table 1).

Analysis of dietary intake revealed significant differences between DII and C‐DII categories for all assessed macronutrients and micronutrients, except for trans fat and the food items garlic and onion. Energy, protein, total fat, carbohydrate, fibre, iron, zinc, magnesium, vitamin A, vitamin C, thiamine, riboflavin, niacin, saturated fatty acids, monounsaturated fatty acids, polyunsaturated fatty acids, cholesterol, vitamin B6, selenium, folate, vitamin B12, vitamin E and vitamin D were significantly higher in the more anti‐inflammatory category (below the median) (*p* < 0.001) for both DII and C‐DII (Table 2).

**TABLE 2:** Dietary intake according to DII and C‐DII categories (*n* = 552) among school‐aged adolescents in Salvador, Bahia, Brazil, 2016–2018.

Dietary parameters	Total	DII			C‐DII		
		Below the median (≤ 2.62) (*n* = 276)	Above the median (> 2.62) (*n* = 276)	*p* ^a^	Below the median (≤ 1.96) (*n* = 276)	Above the median (> 1.96) (*n* = 276)	*p* ^a^
Energy (kcal)	2.033 (727)	2.311.84 (757.56)	1.754.88 (573.14)	0.000	2.315.24 (755.19)	1751.48 (572.93)	0.000
Protein (g)	75 (33)	87.95 (34.2)	61.27 (25.73)	0.000	88.22 (34.07)	61.01 (25.71)	0.000
Carbohydrate (g)	277 (105)	311.97 (111.84)	241.21 (83.10)	0.000	312.21 (111.70)	240.97 (83.09)	0.000
Total fibre (g)	22 (15)	29.31 (17.74)	13.80 (6.28)	0.000	29.39 (17.70)	13.72 (6.1)	0.000
Total fat (g)	70 (32)	79.64 (33.98)	60.61 (27.34)	0.000	79.78 (33.91)	60.47 (27.33)	0.000
Saturated fat (g)	25 (13)	27.65 (13.71)	22.69 (11.49)	0.000	27.69 (13.69)	22.64 (11.50)	0.000
Mono fat (g)	19 (10)	22.82 (11.00)	15.99 (8.55)	0.000	22.88 (10.97)	15.93 (8.54)	0.000
Poli fat (g)	13 (8)	16.04 (7.92)	9.56 (6.30)	0.000	16.04 (7.92)	9.56 (6.30)	0.000
Cholesterol (mg)	217 (143)	259.78 (156.35)	173.27 (112.30)	0.000	260.64 (155.76)	172.41 (112.53)	0.000
Vitamin A (RE)	545 (1.160)	787.61 (158.00)	303.34 (43.00)	0.000	786.86 (435.11)	304.13 (21.35)	0.000
Vitamin B1 (mg)	1.25 (1.02)	1.52 (1.21)	0.97 (0.68)	0.000	1.52 (1.21)	0.97 (0.68)	0.000
Vitamin B2 (mg)	1.48 (1.26)	1.82 (1.55)	1.14 (1.01)	0.000	1.82 (1.39)	1.14 (1.01)	0.000
Vitamin B6 (mg)	1.26 (0.75)	1.58 (0.82)	0.93 (0.49)	0.000	1.58 (0.82)	0.93 (0.49)	0.000
Vitamin B12 (μg)	2.35 (3.96)	3.09 (0.35)	1.61 (1.29)	0.000	3.09 (0.35)	1.60 (1.29)	0.000
Vitamin C (mg)	114 (166)	163.32 (94.47)	64.74 (11.21)	0.000	162.40 (94.43)	65.67 (12.09)	0.000
Vitamin D (μg)	2.45 (2.66)	3.06 (3.01)	1.84 (1.57)	0.000	3.06 (3.01)	1.84 (1.57)	0.000
Vitamin E (mg)	4.9 (5.5)	6.86 (6.43)	2.98 (0.41)	0.000	6.84 (6.44)	2.99 (0.42)	0.000
Niacin (mg)	15 (10)	18.28 (10.99)	11.63 (6.63)	0.000	18.29 (10.98)	11.62 (6.64)	0.000
Folate (μg)	52 (57)	67.22 (9.73)	36.76 (5.47)	0.000	67.15 (9.78)	36.82 (5.44)	0.000
Iron (mg)	9.9 (6.3)	12.51 (7.41)	7.24 (3.37)	0.000	12.54 (7.39)	7.21 (3.37)	0.000
Magnesium (mg)	188 (93)	241.98 (96.64)	134.69 (48.43)	0.000	242.22 (96.47)	134.45 (48.23)	0.000
Selenium (μg)	44 (35)	54.50 (37.61)	32.57 (27.85)	0.000	54.58 (37.54)	32.49 (27.88)	0.000
Zinc (mg)	8.9 (5.2)	10.78 (4.89)	7.05 (4.79)	0.001	10.81 (4.7)	7.01 (4.78)	0.000
Total trans fat (g)	0.35 (0.59)	0.35 (0.57)	0.35 (0.60)	0.920	—	—	—
Onion (g)	0.04 (0.15)	0.04 (0.18)	0.03 (0.11)	0.217	—	—	—
Garlic (g)	0.0118 (0.1345)	0.01 (0.09)	0.01 (0.17)	0.558	—	—	—
Oregano (g)^b^	0	0	0	—	—	—	—
Chilli pepper (g)^b^	0	0	0	—	—	—	—

*Note:* Data are presented as mean (SD); (—) = parameters not used for C‐DII calculation.

Abbreviations: C‐DII, children's dietary inflammatory index; DII, dietary inflammatory index; Mono fat, monounsaturated fat; Poli fat, polyunsaturated fat; SD, standard deviation; Vit, vitamin.

^a^ *p*‐values were calculated for differences between below‐median and above‐median categories of DII and C‐DII using Student's *t*‐test (continuous variables).

^b^ Consumption of these dietary parameters was close to zero.

### 3.2 Dietary Inflammatory Potential (DII and C‐DII) and Cardiometabolic Risk

In the total sample, a positive association was observed between C‐DII and BMI after statistical adjustments. Adolescents with a pro‐inflammatory diet (above the median) showed higher BMI (*β* = 0.97; 95% CI: 0.02–1.92) (Table 3).

**TABLE 3:** Crude and adjusted analyses of associations [beta regression coefficients (95% CI)] between DII and C‐DII categories and cardiometabolic risk indicators in school adolescents in Salvador, Bahia, Brazil, 2016–2018.

Scores		BMI (Kg/m^2^)		WC (cm)		WHtR		SBP (mmHg)		DBP (mmHg)	
		Crude	Adjusted	Crude	Adjusted	Crude	Adjusted	Crude	Adjusted	Crude	Adjusted
DII	Below the median (≤ 2.62) (*n* = 276)	(Ref.)	(Ref.)	(Ref.)	(Ref.)	(Ref.)	(Ref.)	(Ref.)	(Ref.)	(Ref.)	(Ref.)
	Above the median (> 2.62) (*n* = 276)	0.26 (−0.56; 1.08)	0.91 (−0.04; 1.86)	−0.49 (−2.83; 1.84)	0.33 (−2.26; 2.93)	0.01 (0.00; 0.02)	0.01 (0.00; 0.03)	−2.46 (−4.98; 0.05)	−1.18 (−3.96; 1.59)	−0.80 (−2.33; 0.73)	−0.97 (−2.71; 0.76)
C‐DII	Below the median (≤ 1.96) (*n* = 276)	(Ref.)	(Ref.)	(Ref.)	(Ref.)	(Ref.)	(Ref.)	(Ref.)	(Ref.)	(Ref.)	(Ref.)
	Above the median (> 1.96) (*n* = 276)	0.44 (−0.38; 1.26)	0.97 (0.02; 1.92)	−0.60 (−2.93; 1.73)	0.44 (−2.16; 3.04)	0.01 (0.00; 0.02)	0.01 (0.00; 0.03)	−2.44 (−4.95; 0.08)	−1.02 (−3.8; 1.77)	−0.81 (−2.34; 0.72)	−0.89 (−2.64; 0.85)

*Note:* Crude models: dependent variables (BMI, WC, WHtR, SBP, DBP); independent variables (DII and C‐DII). Adjusted model: adjusted for age (continuous), skin colour, physical activity, socioeconomic categories, alcohol consumption and smoking.

Abbreviations: BMI, body mass index; C‐DII, children's dietary inflammatory index; DBP, diastolic blood pressure; DII, dietary inflammatory index; Ref., reference; SBP, systolic blood pressure; WC, waist circumference; WHtR, waist‐to‐height ratio.

When analyses were stratified by sex, adjusted models indicated a positive association between pro‐inflammatory diet and BMI among girls for both indices. On average, girls with a pro‐inflammatory diet (above the median) had BMI values 1.29 kg/m^2^ and 1.38 kg/m^2^ higher than those with an anti‐inflammatory diet (below the median) for DII and C‐DII, respectively. No statistically significant associations were observed for other outcomes, including WC, WHtR, or blood pressure. Among boys, no significant associations were found between the dietary indices and any cardiometabolic indicators (Table 4).

**TABLE 4:** Crude and adjusted analyses of associations [beta regression coefficients (95% CI)] between DII and C‐DII categories and cardiometabolic risk indicators (stratified by sex) in school adolescents. Salvador, Bahia, Brazil, 2016–2018.

Scores		BMI (Kg/m^2^)		WC (cm)		WHtR		SBP (mmHg)		DBP (mmHg)	
		Crude	Adjusted	Crude	Adjusted	Crude	Adjusted	Crude	Adjusted	Crude	Adjusted
Girls											
DII	Below the median (≤ 2.62) (*n* = 276)	(Ref.)	(Ref.)	(Ref.)	(Ref.)	(Ref.)	(Ref.)	(Ref.)	(Ref.)	(Ref.)	(Ref.)
	Above the median (> 2.62) (*n* = 276)	0.44 (−0.62; 1.50)	1.29 (0.13; 2.46)	−1.27 (−4.09; 1.54)	0.90 (−2.36; 4.17)	0.01 (−0.01; 0.02)	0.02 (0.00; 0.03)	−2.14 (−5.54; 1.26)	−0.76 (−4.22; 2.70)	−1.28 (−3.42; 0.86)	−0.97 (−3.28; 1.34)
C‐DII	Below the median (≤ 1.96) (*n* = 276)	(Ref.)	(Ref.)	(Ref.)	(Ref.)	(Ref.)	(Ref.)	(Ref.)	(Ref.)	(Ref.)	(Ref.)
	Above the median (> 1.96) (*n* = 276)	0.48 (−0.58; 1.54)	1.38 (0.21; 2.54)	−1.20 (−4.02; 1.62)	1.05 (−2.23; 4.33)	0.01 (−0.01; 0.02)	0.02 (0.00; 0.03)	−2.02 (−5.42; 1.39)	−0.53 (−4.01; 2.94)	−1.22 (−3.36; 0.92)	−0.85 (−3.17; 1.48)
Boys											
DII	Below the median (≤ 2.62) (*n* = 276)	(Ref.)	(Ref.)	(Ref.)	(Ref.)	(Ref.)	(Ref.)	(Ref.)	(Ref.)	(Ref.)	(Ref.)
	Above the median (> 2.62) (*n* = 276)	−0.17 (−1.45; 1.12)	−0.02 (−1.54; 1.50)	1.68 (−2.53; 5.90)	−1.33 (−5.42; 2.77)	0.01 (−0.01; 0.03)	0.00 (−0.02; 0.03)	−2.00 (−5.28; 1.28)	−2.26 (−6.62; 2.11)	−0.41 (−2.20; 1.30)	−0.84 (−3.10; 1.42)
C‐DII	Below the median (≤ 1.96) (*n* = 276)	(Ref.)	(Ref.)	(Ref.)	(Ref.)	(Ref.)	(Ref.)	(Ref.)	(Ref.)	(Ref.)	(Ref.)
	Above the median (> 1.96) (*n* = 276)	0.29 (−1.00; 1.57)	−0.02 (−1.54; 1.50)	1.21 (−3.01; 5.44)	−1.33 (−5.42; 2.77)	0.01 (−0.01; 0.03)	0.00 (−0.02; 0.03)	−2.02 (−5.31; 1.27)	−2.26 (−6.62; 2.11)	−0.55 (−2.34; 1.24)	−0.84 (−3.10; 1.42)

*Note:* Crude models: dependent variables (BMI, WC, WHtR, SBP, DBP); independent variables (DII and C‐DII). Adjusted models: adjusted for age (continuous), skin colour, physical activity, socioeconomic categories, alcohol consumption and smoking.

Abbreviations: BMI, body mass index; C‐DII, children's dietary inflammatory index; DBP, diastolic blood pressure; DII, dietary inflammatory index; Ref., reference; SBP, systolic blood pressure; WC, waist circumference; WHtR, waist‐to‐height ratio.

When models were stratified by age (Table 5), adolescents older than 14 years consuming a pro‐inflammatory diet (above the median) had an adjusted mean BMI 2.16 kg/m^2^ higher (95% CI: 0.62–3.71) compared with those consuming an anti‐inflammatory diet (below the median) for both DII and C‐DII. Similarly, WC was on average 3.73 cm greater (95% CI: 0.24–7.23) and WHtR was 0.03 higher (95% CI: 0.01–0.05) in adolescents with a pro‐inflammatory diet, with identical values for both indices. No statistically significant associations were observed between the indices and blood pressure in this age group. Among adolescents aged ≤ 14 years, no significant associations were found between the dietary indices and any cardiometabolic indicators in either model.

**TABLE 5:** Crude and adjusted analyses of associations [beta regression coefficients (95% CI)] between DII and C‐DII categories and cardiometabolic risk indicators (stratified by age group) in school adolescents in Salvador, Bahia, Brazil, 2016–2018.

Scores		BMI (Kg/m^2^)		WC (cm)		WHtR		SBP (mmHg)		DBP (mmHg)	
		Crude	Adjusted	Crude	Adjusted	Crude	Adjusted	Crude	Adjusted	Crude	Adjusted
≤ 14 anos											
DII	Below the median (≤ 2.62) (*n* = 276)	(Ref.)	(Ref.)	(Ref.)	(Ref.)	(Ref.)	(Ref.)	(Ref.)	(Ref.)	(Ref.)	(Ref.)
	Above the median (> 2.62) (*n* = 276)	0.13 (−0.91; 1.18)	0.27 (−0.96; 1.50)	−1.01 (−4.22; 2.20)	−1.10 (−4.77; 2.56)	0.00 (−0.0.1; 0.01)	0.00 (−0.01; 0.02)	−2.46 (−6.01; 1.09)	−2.45 (−6.24; 1.34)	−1.40 (−3.59; 0.80)	−1.92 (−4.40; 0.55)
C‐DII	Below the median (≤ 1.96) (*n* = 276)	(Ref.)	(Ref.)	(Ref.)	(Ref.)	(Ref.)	(Ref.)	(Ref.)	(Ref.)	(Ref.)	(Ref.)
	Above the median (> 1.96) (*n* = 276)	0.19 (−0.86; 1.24)	0.36 (−0.87; 1.59)	−0.91 (−4.12; 2.30)	−0.96 (−4.64; 2.71)	0.00 (−0.0.1; 0.01)	0.01 (−0.01; 0.02)	−2.32 (−5.87; 1.23)	−2.21 (−6.01; 1.59)	−1.31 (−3.51; 0.89)	−1.80 (−4.29; 0.68)
> 14 anos											
DII	Below the median (≤ 2.62) (*n* = 276)	(Ref.)	(Ref.)	(Ref.)	(Ref.)	(Ref.)	(Ref.)	(Ref.)	(Ref.)	(Ref.)	(Ref.)
	Above the median (> 2.62) (*n* = 276)	1.20 (−0.08; 2.48)	2.16 (0.62; 3.71)	2.60 (−0.55; 5.74)	3.73 (0.24; 7.23)	0.02 (0.00; 0.04)	0.03 (0.01; 0.05)	0.10 (−2.95; 3.14)	1.42 (−2.50; 5.34)	1.44 (−0.34; 3.22)	0.94 (−1.24; 3.12)
C‐DII	Below the median (≤ 1.96) (*n* = 276)	(Ref.)	(Ref.)	(Ref.)	(Ref.)	(Ref.)	(Ref.)	(Ref.)	(Ref.)	(Ref.)	(Ref.)
	Above the median (> 1.96) (*n* = 276)	1.61 (0.34; 2.90)	2.16 (0.62; 3.71)	2.32 (−0.83; 5.48)	3.73 (0.24; 7.23)	0.02 (0.00; 0.03)	0.03 (0.01; 0.05)	0.15 (−2.90; 3.19)	1.42 (−2.50; 5.34)	1.40 (−0.38; 3.19)	0.94 (−1.24; 3.12)

*Note:* Crude models: dependent variables (BMI, WC, WHtR, SBP, DBP); independent variables (DII and C‐DII). Adjusted models: for sex, skin colour, physical activity, socioeconomic categories, alcohol consumption and smoking.

Abbreviations: BMI, body mass index; C‐DII, children's dietary inflammatory index; DBP, diastolic blood pressure; DII, dietary inflammatory index; Ref., reference; SBP, systolic blood pressure; WC, waist circumference; WHtR, waist‐to‐height ratio.

## 4 Discussion

This study investigated the association between dietary inflammatory potential, assessed by the DII and C‐DII and cardiometabolic risk indicators in a sample of adolescents from public schools in Salvador, Bahia, Brazil. Overall, the findings indicated that a pro‐inflammatory diet was associated with higher adiposity levels in girls, as evidenced by higher mean BMI. Additionally, among adolescents older than 14 years, these effects extended to indicators of abdominal obesity, including WC and WHtR. In contrast, no significant associations were observed with blood pressure in any of the models, regardless of sex or age group.

Mean DII (2.44) and C‐DII (1.78) scores observed in the adolescents of this study indicate a predominantly pro‐inflammatory diet, with values higher than those reported in previous studies [23, 24, 52, 53]. In Brazil, earlier research reported lower mean DII scores, such as 1.71 in adolescents from São Luís [24] and 1.04 in the 2008–2009 Household Budget Survey (POF) [53]. Internationally, even lower values have been observed, including a C‐DII of 0.23 in the Avon Longitudinal Study of Parents and Children (ALSPAC) [52] and a DII of −1.46 in the National Health and Nutrition Examination Survey (NHANES) [23]. These findings suggest that the adolescents evaluated are more exposed to pro‐inflammatory dietary patterns, potentially related to higher consumption of ultra‐processed foods and lower intake of fruits, vegetables and whole foods, as previously identified in a study with the same sample [12].

Previous studies investigating the dietary inflammatory potential in adolescents have reported findings consistent with those observed in the present study [49, 54, 55]. In Brazil, for example, Blaudt [54] and Todendi et al. [49], using data from the Study of Cardiovascular Risk in Adolescents (ERICA), identified significant associations between pro‐inflammatory diets (assessed by DII) and excess weight, abdominal obesity, as well as alterations in glycemic parameters. However, these associations were observed in both sexes, unlike the present study, which found significant associations only in girls. Similarly, in Zhang et al., adolescents (mean age = 15.9 years) in the third DII quartile had a higher risk of overweight/obesity (OR = 1.46; 95% CI: 1.24–1.71) after adjustments. Regarding central obesity, adolescents in the highest DII quartile had an increased risk, independent of demographic characteristics and physical activity [55].

Other studies further support this association. Jia et al. [23], Kurklu Seremet et al. [22] and Vahid et al. [56] reported significant relationships between high DII scores and various cardiometabolic risk markers in adolescents, including excess weight and abdominal obesity, although these studies did not stratify by sex or age. These findings are consistent with the results presented here and reinforce the hypothesis that the dietary inflammatory profile is a sensitive marker for identifying early trends of cardiometabolic deterioration during adolescence [22, 23, 49, 54, 55, 56], potentially increasing the risk of developing non‐communicable chronic diseases in adulthood [17, 57].

Although less explored in the literature, some studies have applied the C‐DII, but results remain heterogeneous [52, 58, 59, 60, 61]. For example, Aljahdali et al., assessing healthy adolescents, found no positive association between C‐DII and WC or blood pressure and no sex differences were observed [59]. In contrast, Sethna et al., using NHANES data, reported that adolescents with higher C‐DII scores had an increased risk of central obesity [60]. Conversely, Ducharme‐Smith et al., analysing U.S. adolescents, observed an association between C‐DII and a more favourable lipid profile [61]. In the study conducted by Çağiran Yilmaz & Açık, C‐DII was modestly directly associated with fasting insulin, fasting blood glucose and waist circumference [62].

It is important to note that this study demonstrated that the effects of a pro‐inflammatory diet may become more evident as adolescents age (> 14 years), likely due to longer exposure and hormonal, metabolic and physiological changes typical of puberty [63]. Buckland et al. also identified that higher C‐DII during childhood (7 years) and at 13 years was associated with increases in BMI, fat mass, blood pressure and insulin resistance during adolescence (17 years) [52]. These developmental changes affect body fat accumulation and distribution, increasing vulnerability to inadequate dietary patterns, whereas in early adolescence the immaturity of hormonal and metabolic systems may provide temporary protection [63, 64, 65].

Furthermore, the association between a pro‐inflammatory diet and adiposity was observed only in girls, particularly when assessed by BMI, which differs from some previous studies [54, 59]. This finding may be partly explained by sex‐specific biological and behavioural factors. For instance, hormonal changes during puberty, such as increases in oestrogen levels, may favour subcutaneous fat deposition and potentially influence sensitivity to dietary factors; however, as pubertal stage was not assessed in this study, this interpretation should be interpreted with caution [63, 64, 65]. Behavioural and social factors also play an important role, as adolescents of different sexes exhibit distinct dietary patterns, with girls in some contexts consuming more ultra‐processed foods and being more concerned with body image, which can influence their food choices [66].

Regarding blood pressure, although some studies have reported associations between pro‐inflammatory diets and increased blood pressure in adolescents [23, 49, 62, 67], the present study found no significant relationship between dietary inflammatory indices and blood pressure. While this absence may be partly attributable to the cross‐sectional design, which is unable to capture the cumulative effects of diet [67], physiological mechanisms should also be considered [68, 69]. Blood pressure alterations in adolescents may manifest only after prolonged exposure to risk factors [52], given the greater vascular plasticity and adaptive capacity at this age. Additionally, hormonal changes related to puberty, rapid linear growth and inter‐individual variability in sodium sensitivity may attenuate the immediate detection of dietary effects on blood pressure [68, 69].

Another relevant finding of this study was the similar performance of DII and C‐DII, indicating that, in the analysed sample, both indices exhibited virtually identical behaviour in relation to cardiometabolic outcomes. This may reflect low dietary inflammatory variability or methodological limitations of the indices themselves. The C‐DII is a recent paediatric adaptation, based on adult data and a reduced number of dietary parameters. For the DII, only 28 of the 45 originally recommended parameters were used, which may have contributed to the similarity between the indices [14, 57]. Therefore, in this study, both indices were constructed using largely overlapping nutrients and their dichotomisation may have further contributed to their comparable performance. Nevertheless, the C‐DII appeared to perform slightly better with BMI compared to DII when the sample was not stratified by sex and age; however, these differences were minimal and are unlikely to be clinically meaningful.

This study has some limitations. The absence of significant associations for some variables may be attributed to several factors, including potential homogeneity of dietary patterns among participants, which could reduce the variability needed to detect associations. In addition, inflammatory dietary habits may exert cumulative effects over time, becoming more evident in later life stages. Moreover, DII and C‐DII scores in this study were calculated based on 28 and 23 dietary parameters, respectively, instead of the full set of 45 or 25 items in the original indices [14, 25]. The use of a limited number of components may affect comparability with findings from studies using the complete set of parameters and could have influenced the magnitude of the observed associations.

Additionally, participants were recruited from both military and public schools using convenience sampling, which may limit the generalisability of the findings. These school environments may differ systematically in factors such as physical activity patterns, discipline and socioeconomic characteristics, potentially influencing the observed associations. Furthermore, abdominal obesity was defined using the 90th percentile of the study sample, which may limit comparability with other studies that adopt external reference standards. However, in the association analyses, adiposity indicators were primarily analysed as continuous variables, which may reduce potential bias related to this classification. A final limitation is the possibility of residual confounding, as, despite adjustment for variables identified in an extensive literature review, unmeasured factors such as pubertal stage and sleep quality were not included.

Despite these limitations, it is important to highlight that the present study incorporated a robust set of control variables, including sociodemographic and behavioural factors, which enhances the rigour of the analyses. Another methodological strength was the use of the Multiple Source Method (MSM) to estimate usual dietary intake, correcting for intra‐individual variability and improving the accuracy of DII and C‐DII scores—a particularly relevant approach in adolescent populations, where dietary intake tends to be more irregular. It should also be noted that some dietary parameters, such as turmeric, ginger, teas and other items not included in the calculation, are rarely or never consumed by the study population. Furthermore, the multilevel modelling approach accounted for the hierarchical data structure (schools as clustering units), reducing potential school‐related biases. Finally, this is the first study to use the C‐DII in a Brazilian adolescent population and to evaluate its performance alongside the DII, assessing potential differences between the indices and providing additional evidence for their applicability.

## 5 Conclusion

The results of this study indicate that diets with higher inflammatory potential, assessed using both indices, are associated with higher adiposity levels in adolescents, particularly among girls and those older than 14 years, as evidenced by BMI, WC and WHtR. Conversely, no significant associations were observed with blood pressure, suggesting that dietary effects on this outcome may manifest later in life or be modulated by a combination of behavioural, genetic, or environmental factors.

These findings highlight the importance of future longitudinal studies incorporating repeated dietary assessments, inflammatory biomarkers and blood pressure measurements to elucidate the mechanisms involved and the timing of deleterious effects of an inflammatory diet. Additionally, it is essential that the indices used are validated across different cultural contexts and pubertal development stages. Finally, early nutritional interventions—particularly in school settings—focusing on promoting healthier and less inflammatory dietary patterns during adolescence are crucial strategies for preventing chronic diseases and promoting cardiometabolic health throughout life.

## Author Contributions

L.O.L. and P.R.F.C. designed the study. L.O.L. and P.R.F.C. drafted the manuscript and conducted critical revisions. L.O.L. and N.F.D. performed data collection, coding and interpretation of dietary intake. L.O.L., P.R.F.C., M.L.P.S. and J.C.D.P. analysed and interpreted the data. All authors read, contributed to the manuscript writing and approved the final version.

## Funding

The authors have nothing to report.

## Conflicts of Interest

The authors declare no conflicts of interest.

## Data Availability

The data that support the findings of this study are available on request from the corresponding author. The data are not publicly available due to privacy or ethical restrictions.
